# Barriers and facilitators to engaging in a university-based exercise programme delivered to students experiencing mental health difficulties: A pilot study

**DOI:** 10.1080/28324765.2024.2380500

**Published:** 2024-07-31

**Authors:** Gary Skinner, Megan Teychenne, Joey Murphy

**Affiliations:** a Centre for Exercise, Nutrition and Public Health, School for Policy Studies, University of Bristol, Bristol, UK; b Institute for Physical Activity and Nutrition (IPAN), School of Exercise and Nutrition Sciences, Deakin University, Geelong, Victoria, Australia

**Keywords:** Mental health, tertiary students, exercise, exercise referral scheme, qualitative research, tertiary education

## Abstract

Prevalence of mental health problems among university students is high. Exercise can benefit mental health and universities could provide suitable settings for delivering exercise referral schemes (ERS). This study examined factors influencing exercise engagement among participants of a university-based ERS for mental health. Semi-structured interviews captured the experiences of university students (*n* = 7) experiencing mental health difficulties who had participated in a university-based ERS. Instructors participated in a focus group to further assess the factors influencing exercise engagement. Data was analysed using reflexive thematic analysis. Twelve themes were constructed and organised using the COM-B model which describes the interaction between Capability, Opportunity and Motivation to generate a particular behaviour. The Behaviour in this study is considered exercise. Themes were categorised in the following way: Capability (experience and knowledge, anxiety, skills, and physical fitness and health), Opportunity (accessibility, time, social support, and subjective norm), and Motivation (planning and routine, goal-setting, benefits of exercising, and enjoyment). Practitioners implementing university-based exercise referral schemes to support mental health may consider these learnings to help support the capability, opportunity and motivation of students to engage and remain engaged in exercise. Further research is recommended to assess feasibility of similar programmes in other universities or settings.

## Introduction

Mental ill-health affects one-in-four people annually including conditions such as depression, anxiety, panic disorder, phobias and obsessive-compulsive disorder (Baker & Kirk-Wade, 2023; Mind, 2022). In 2010, it was estimated that poor mental health cost the global economy $2.5 trillion annually, with this projected to increase to 6 trillion by 2030 (The Lancet Global Health, 2020). The period of young adulthood, especially through university, is a transitionary period often met with stressors related to social situations, loneliness, academic and financial pressures (Duffy et al., 2019). As such, mental ill-health may have a negative impact on student wellbeing and academic performance (Duffy et al., 2020). According to a 2021 systematic review and meta-analysis including studies from various countries, the COVID-19 pandemic has had a negative impact on the mental health of university students, with the prevalence of anxiety and depression reaching 36% and 39% respectively (Li et al., 2021). This is significantly higher than the 16% reported for the general UK adult population (Office for National Statistics, 2022).

Exercise is a sub-set of physical activity where bodily movement is planned, structured and repetitive (Caspersen et al., 1985). Physical activity is associated with enhanced wellbeing independent of age and thus, exercise can be used as an adjunct treatment or preventative option for improved mental health outcomes (Marconcin et al., 2022; Mikkelsen et al., 2017). When considering Hettler’s model that contains six dimensions of wellness, according to the physical domain, regular exercise may subsequently lead to psychological benefits in the form of enhanced self-esteem, self-control, determination and a sense of direction (National Wellness Institute, 2023b). Thus, interventions incorporating exercise may be effective in supporting university students with mental health difficulties as long as they are tailored to individual health, age, social situation and physical activity interests (Huang et al., 2018; Lange, 2018). For optimal physical and mental health, those aged 19–64 are advised to do muscle strengthening activities at least twice weekly and accumulate 150 weekly minutes of moderate aerobic activity or 75 minutes when performing more vigorous activity (Department for Health and Social Care, 2022). Both aerobic and resistance exercise can benefit mental health by improving symptoms including stress, anxiety and depression (Aylett et al., 2018; Gordon et al., 2017, 2018; Mikkelsen et al., 2017; Morres et al., 2019; Portugal et al., 2013; Smith & Merwin, 2020). Evidence suggests this inverse association also exists for young adults and university students (Dogra et al., 2018; Gerber et al., 2014; Herbert et al., 2020; Murphy et al., 2018; Song et al., 2021; Tyson et al., 2010; von Haaren et al., 2015). As such, exercise has demonstrated improvements in both the physical and mental health of university students which can benefit academic outcomes (Bruffaerts et al., 2018; Redondo-Flórez et al., 2022; Rodríguez-Romo et al., 2023).

In order to obtain the long-term mental health benefits of exercise, engagement should be sustained over time (Smith & Merwin, 2020). Therefore, understanding the barriers and facilitators to exercise adherence are important to ensure engagement can be achieved and maintained over a sustained period. Facilitators for exercise among university students reported in the literature include self-discipline, prior exercise exposure, social support and a selection of enjoyable activities (Deliens et al., 2015; Newsome et al., 2021; Silver et al., 2019). Meanwhile, barriers reported include time constraints, academic commitments, inadequate accessibility, cost, feeling incompetent and perceived physical exertion (2006; Daskapan et al., Deliens et al., 2015; Gómez-López et al., 2010; Mohamad Nizam Nazarudin et al., 2018; Newsome et al., 2021; Silva et al., 2022). Those experiencing mental distress may have unique facilitators and barriers that need to be considered for effective exercise interventions (Rebar & Taylor, 2017). Therefore, it is important to acknowledge the potential facilitators and barriers university students with mental health difficulties may face when seeking to engage in exercise and participate in exercise programmes.

There is limited research about exercise programming for mental health among university students and this context may offer a feasible setting to better understand exercise as a mental health management approach (deJonge et al., 2020). Recent evidence suggests the potential feasibility and acceptability of exercise services among university students with mental health difficulties but more research on the elements of such programmes can help to better understand the implications on engagement (Jeftic, et al., 2023a). As such, there is a need for studies focusing on the “individual person” in terms of needs, preferences, motivation and reasons for engaging in exercise (Herbert, 2022). This is especially important to consider when prescribing exercise for mental health as the benefits of recreational physical activity for mental health are likely to be enhanced through personal enjoyment (Teychenne et al., 2020; Vella et al., 2023).

An Exercise Referral Scheme (ERS) can help participants increase physical activity and subsequently, obtain the health benefits associated with exercise (National Institute for Health and Care Excellence, 2022). Therefore, an ERS can be utilised in universities to aid the mental wellbeing of students. Presently, exercise programmes for mental health are not yet common in university campuses and further evidence to inform their development may lead to more optimal outcomes (Jeftic, et al., 2023b). This means further research is required to determine the potential barriers and facilitators to engagement in such programmes amongst students who may be experiencing mental health difficulties. It is important to understand the facilitators and barriers for maintaining exercise in order to increase exercise adherence which can subsequently improve the health of those who engage in exercise interventions for mental wellbeing (Sunesson et al., 2021).

The primary aim was to identify the perceived facilitators and barriers to engaging in exercise faced by university students with mental health difficulties. The objective of this study was to investigate this aim through the lens of a university-based exercise referral programme and assess its role in facilitating exercise participation among participants. These findings may inform the development of future university-based exercise programmes to support the mental health of students.

## Methods

### Study design

This was a qualitative study with ethical approval granted by the School for Policy Studies Research Ethics Committee at the University of Bristol (reference number: 10982).

### The healthy minds programme

Healthy Minds is a 12 week ERS based in the University of Bristol’s indoor sports centre in which students receiving support from the Student Counselling Service or the Mental Health Advisory Service may be eligible (Healthy Minds - Journey, 2023; University of Bristol, 2024). Therefore, this ERS is specifically designed for students who are facing an array of mental health challenges and aims to improve participant wellbeing through exercise. The programme is free of charge with participants granted full access to university exercise facilities such as the gym and swimming pool. In addition, participants are able to attend the fitness class offering in the indoor sports centre that can be utilised in their own time. Similar to other exercise referral schemes for health conditions, participants are also offered face-to-face supervised exercise sessions with an instructor which can incorporate aerobic and resistance exercise (Rowley et al., 2018). These formal sessions with a qualified fitness instructor occur at least every four weeks for about an hour and typically take place at the University of Bristol’s indoor sports centre. Participants can liaise with the instructor to arrange sessions at an appropriate time but also have access to their instructor throughout the duration of the programme in the form of communication, advice and exercise programming guidance. At the beginning of the programme, participants are encouraged to set personal goals by the instructors. Thus, the role of the instructors is to facilitate the achievement of such goals through working with the participant and promoting exercise. The programme tries to find out what forms of exercise participants enjoy to ensure each activity programme is bespoke in nature to cater for individual preferences, needs and circumstance (University of Bristol, 2022). Therefore, the programme provides the opportunity for tailored activity which is consistent with recommendations outlined in a systematic review (Tomlinson-Perez et al., 2022). This is important given that elements of enjoyment and personal preference/autonomy are particularly important to enhance the mental health benefits of physical activity (Teychenne et al., 2020).

### Participants

#### Inclusion criteria

Phenomenology was utilised due to its focus on the lived experiences of individuals (Bowling & Ebrahim, 2005). This was appropriate for this study as our aim was to understand the perceived facilitators and barriers to engaging in exercise among those who participated in a university-based ERS for mental health. Therefore, criterion sampling was used to ensure participants met pre-defined criteria in order to participate in the study (Moser & Korstjens, 2018). This required the recruitment of university students at the University of Bristol, who had involvement in the Healthy Minds programme. All participants were aged 18 years or older and were either currently involved in Healthy Minds at the time of the study (June 2022) or had completed the programme during the 2021/2022 academic year. Students who presented to the Student Counselling Service or the Mental Health Advisory Service and were identified at that point as experiencing mental health difficulties were referred to the Healthy Minds programme. An additional one-month access to the programme was offered to participants as compensation for their time. All participants provided informed consent in order to participate in the study.

#### Sample and recruitment

Sample size was determined using the concept of Information Power, which is useful when aiming to utilise reflexive thematic analysis (Braun & Clarke, 2019). Information Power indicates that if a group of participants hold a large amount of information relevant for the study, a smaller sample size is suitable and is considered by looking at the following dimensions: (a) study aim, (b) sample specificity, (c) use of established theory, (d) quality of dialogue, and (e) analysis strategy (Malterud et al., 2016).

- Although a broad aim requires a larger sample, when the aim incorporates a specific experience, the number of eligible participants is limited (Malterud et al., 2016). This study aimed to investigate the experiences of university students enrolled in Healthy Minds, which limited the scope of sampling.
- A less extensive sample is required when participants hold characteristics that are specific for the research aim (Malterud et al., 2016). In this case, all participants belonged to a specific target group while also providing variation in relation to their experience of the research phenomenon.
- Although the planning of this study was not rooted in theory, the subsequent analysis became grounded in the COM-B model of behaviour. Thus, empirical research with a small sample can make a difference if something pivotal to theory is addressed (Malterud et al., 2016).
- A richer quality of dialogue typically requires fewer participants (Malterud et al., 2016). The dialogue was deemed to be strong, which is supported with quotes throughout the results section and the inclusion of a meaning units table in supplementary materials. This shows similar concepts were discussed in each interview which indicated rich information being gathered that was relevant to the research aim. Additionally, it shows how the views offered by the instructors aligned with those presented by the participants.
- Reflexive thematic analysis was used to provide an in-depth examination of participants’ experiences providing rich information which requires insights from a few, selected participants (Malterud et al., 2016). Details of the analysis approach are presented later in this section.

The gatekeeper (Healthy Minds supervisor) sent a recruitment email for the study to 65 students who met eligibility criteria. Posters were also placed in the University of Bristol’s indoor sports centre to increase awareness of the study among potential participants. Seven participants consented to participate in individual interviews. This was considered suitable as fewer than 10 interviews are appropriate for phenomenological studies (Moser & Korstjens, 2018). Furthermore, three Healthy Minds instructors were invited to participate in a focus group discussion to offer their experiences of the programme. The focus group provided an additional source of data which enhanced rigor by confirming the experiences offered by student participants through triangulation (Johnson et al., 2020). It was viewed as important to include and consider the insights of staff who deliver such programmes because they are also key stakeholders when seeking to implement interventions in real-world settings (Koorts et al., 2018). Based on the different dimensions of Information Power, and the fact data generated was sufficiently rich enough, the sample size of ten (seven participants and three instructors) was deemed enough to address the aims of this research study.

### Data collection

#### Topic guides

When collecting data via individual interviews and focus group discussion, the objective was to utilise topic guides for each context that ensured rigor, objectivity and trustworthiness (Kallio et al., 2016). Both topic guides were created by the first author and then reviewed by the third author and the Healthy Minds supervisor. This ensured all questions were relevant to address the research aims and clear for the target population. Both guides were then pilot-tested (interview topic guide with a university student and focus group topic guide with the Healthy Minds supervisor) to determine flow (Bryman, 2012). The individual interview topic guide examined the participants’ perceptions of exercise and their journey through Healthy Minds. Therefore, seven overarching questions containing multiple prompts were asked that covered perceptions of physical activity and wellbeing, motivation to engage in exercise, enjoyment of exercise, format and components of the Healthy Minds programme, adherence to the Healthy Minds programme, facilitators and barriers to engaging in exercise, effects of Healthy Minds in terms of exercise adherence, wellbeing and plans for sustained exercise engagement post programme. These questions were seen as appropriate to address the research aims within a semi-structured format in order to generate strong dialogue required to obtain sufficient information power (Adeoye-Olatunde & Olenik, 2021; Malterud et al., 2016). For example, to explore the barriers and facilitators to engagement and adherence to exercise in general, participants were asked questions such as “Do you enjoy exercising or see it as a chore?” and “What are your perceptions of the gym environment?”. To explore factors related to engagement and adherence to the Healthy Minds programme specifically, participants were asked questions such as “Did you find it manageable to attend sessions when aiming to balance other commitments and time-constraints?” and “Do you believe the programme has helped tackle any potential barriers to exercise that previously existed for you?”. Furthermore, to identify elements of the programme requiring modification and strategies to enhance exercise facilitation, questions such as “Do you think any adjustments should be made to the programme structure or offerings?” were asked.

Meanwhile, the focus group topic guide enabled the interviewer to ask about what the instructors perceive to be the facilitators and barriers to exercise faced by participants based on their experience of working with participants over recent years. The focus group discussion also provided the opportunity for instructors to offer their own perceptions of Healthy Minds and potential suggestions for refinement. For example, instructors were asked: “Are there any adjustments to the present Healthy Minds structure you would make?”. Topic guides are included in the supplementary materials.

#### Interviews and focus group

One-to-one interviews with Healthy Minds participants occurred via Zoom during the month of June 2022. The average duration was 31 minutes. Individual interviews were deemed suitable especially when discussing potentially sensitive topics such as mental health (Minhat, 2015). Conducting the interviews online provided flexibility and removed any barriers related to travel, helping participants to engage in the study (Gray et al., 2020). As mentioned, the subsequent utilisation of a focus group discussion provided an additional data source and allowed insights to be gained from the instructors to support interview findings. A focus group was deemed appropriate for the instructors because this format typically suits a cohort who are comfortable with each other (Isaacs, 2014). The focus group also took place on Zoom and lasted 45 minutes. This occurred in July 2022, after individual interviews were completed with participants. Both the interviews and focus group were audio and visually recorded, and transcribed via Zoom. However, a back-up audio recording device (Apple iPhone 11) was utilised to avoid losing any audio-recorded data. In line with participant confidentiality, all identifying information was removed from transcripts with participants assigned a number (Korstjens & Moser, 2017).

### Data analysis

Reflexive thematic analysis was utilised in order to identify coherent patterns present in the data (Braun & Clarke, 2023; The University of Auckland, 2023). As per the guidance of Braun and Clarke, six phases were followed when analysing the data (Braun & Clarke, 2006, 2023; Braun et al., 2019; The University of Auckland, 2023).

1. **Familiarisation**: To ensure sufficient familiarity with the data, interview and focus group transcripts were read multiple times. These transcripts were generated via zoom. By listening to the recordings, transcripts were corrected to have an accurate record of what was said by participants. The transcript was subsequently downloaded and placed into Microsoft Word. Data was then anonymised and re-examined to ensure confidentiality and transcription accuracy. In addition, interviews were watched repeatedly to facilitate note taking.
2. **Generating codes**: Using NVivo, the first author generated initial codes using an inductive approach (Braun & Clarke, 2006). These codes acted as labels that described relevant aspects of the data. The codes created were semantic in nature (Braun et al., 2019). Multiple rounds of coding were conducted by the first author before they were subsequently reviewed by the third author to ensure clarity and aid the reflexive process throughout the data analysis.
3. **Constructing themes**: Common codes were grouped into potential themes, and although the initial coding was inductive, certain themes aligned with the COM-B model of behaviour by fitting the scope within each domain of the model. This model describes the interaction between capability, opportunity and motivation to generate behavioural outcomes (Michie et al., 2011). The COM-B model was acknowledged for subsequent phases of data analysis as this enabled the authors to convey the data in a coherent manner relevant to the research objective (Braun et al., 2019).
4. **Reviewing themes**: Both the first and third authors reviewed the themes to make necessary refinements. This ensured the themes clearly reflected the data collected. This phase further emphasised COM-B’s relevance to aspects of the data, which is useful for predicting physical activity behaviour (Howlett et al., 2019). Therefore, the authors determined this model to be applicable and suitable for conveying the facilitators and barriers to exercise as shown in Figure 1.

**Figure 1.**
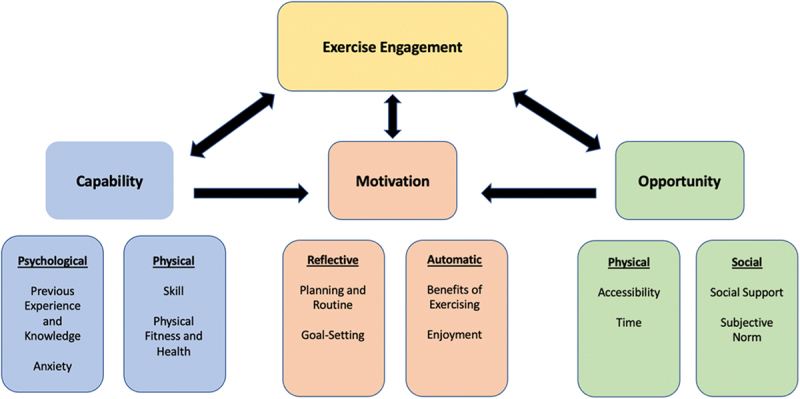
Exercise facilitators and barriers conveyed through COM-B.
5. **Defining themes**: This phase involved establishing the scope and focus of each theme (The University of Auckland, 2023). Factors influencing exercise engagement and adherence were organised using the COM-B model, highlighting perceived facilitators and barriers experienced by participants. A form of thematic mapping was utilised to visualise how themes would fit together by not overlapping (Braun et al., 2019).
6. **Producing the report**: The final phase confirmed to the three authors of this study that the themes worked together to accurately and fully reflect the obtained data. The objective of this phase was to tell the story of the data and its relatedness to the research aim (Braun & Clarke, 2006).

### Reflexivity

It is important that a researcher’s position is transparent where one is aware of potential biases and assumptions to ensure the fair judgement of obtained data (Reid et al., 2018). Therefore, to increase rigour of the above methods, reflexivity was used throughout to ensure self-awareness of the first author while conducting each interview (Johnson et al., 2020). This approach was implemented by creating a reflexive piece and supplementing interviews with reflexive notes (Korstjens & Moser, 2018). The first author conducted all interviews with participants and the focus group with the instructors. This author was formally an instructor in Healthy Minds which ensured the interviewer had pre-existing knowledge of the programme and thus, was able to draw out in-depth information from participants. However, it must be noted that the interviewer instructed one of the study participants. At the time of the study, the first author was a Masters Student in the area of Public Health, had a personal training qualification and had previously competed in high-level sport. This meant the author also had some pre-existing knowledge and understanding of the potential facilitators and barriers that may influence participation in exercise. The second and third authors were both external researchers to Healthy Minds and measures were in place to help mitigate potential bias in the research methods and findings. The third author reviewed the interview and focus group questions to ensure the first author didn’t use leading phrases to help ensure participants honest reflections were captured. The second and third authors reviewed the themes to ensure that the themes identified were clear, but also supported by quotes from the interviews. Therefore, the second author had the opportunity to review the themes generated from the data analysis but was not directly involved in participant data collection and the analysis.

## Results

### Sample description

Seven Healthy Minds participants participated in the study, with characteristics available in Table 1. Participants identified as male (*n* = 2) and female (*n* = 5), aged between 20–29 years (mean = 23 years). All were enrolled at the University of Bristol. Three instructors participated in the focus group discussion. All instructors identified as female with two instructors reporting five years’ experience with Healthy Minds (Instructors 1 and 2) and one reporting three-to-four years (Instructor 3).

**Table 1.** Description of participants

	Age	Gender	Course of Study	Year of Study
Participant 1	29	Female	Law*	2
Participant 2	21	Male	Economics	2
Participant 3	21	Female	Language Studies	4
Participant 4	22	Female	Accelerated Veterinary Medicine	1
Participant 5	23	Female	International Security*	4
Participant 6	20	Female	Law	1
Participant 7	25	Male	Aerospace Engineering	3

*denotes a Masters course. All others are Undergraduate courses.

### Facilitators and barriers to exercise engagement

Facilitators and barriers to exercise engagement, as perceived by this population was organised based on the three core areas of the COM-B model. These are described below and presented in Figure 1. Within each of theme, the role Healthy Minds played in overcoming barriers and suggestions to potentially enhance exercise engagement among participants were identified. Additional meaning units are included in supplementary materials.

### Capability

In our study, factors relating to psychological (experience and knowledge, anxiety) and physical (skills, physical fitness and health) capability were perceived to influence exercise engagement.

### Experience and knowledge

Participants’ knowledge and previous experience of an exercise environment was found to be an important factor for exercise engagement. Having insufficient knowledge and limited prior exposure to gym settings was seen to create barriers, especially when there are multiple pieces of equipment to navigate in fitness environments such as the gym:

“You kind of feel a little bit inferior going there for the first time and using machines that you don’t necessarily know what you’re doing”. (Participant 1)

Having sufficient prior exposure to a gym setting was seen to facilitate exercise engagement, however the format of exercise could also create a barrier:

I never really experienced any challenges because I was familiar with the gym because I’ve been going to the gym for a while. I’d say one challenge I did experience was using fitness classes because I’ve never really gone to fitness classes. (Participant 2)

### Anxiety

Participants reported feeling anxious and intimidated while exercising in certain contexts. This included the fear of certain forms of exercise: “I’ve avoided things like spinning classes because they scare me” (Participant 5), and the feeling of being watched or judged by others, especially when the gym is busy: “Just as an anxious person in general, when there’s loads of people around, you kind of feel like you’re being looked at or judged” (Participant 3). The instructors confirmed this insight by noting anxiety as a barrier. They also discussed self-consciousness and how some participants have this feeling when unsure how to perform exercises correctly compared to others:

“I feel like people are very scared of going into the gym because they think everybody like knows everything what they’re doing”. (Instructor 3)

Having the one-to-one sessions can alleviate this concern as instructors can support participants by demonstrating how to correctly perform certain exercises. Thus, increasing the frequency of instructor-led sessions at the beginning of the programme could be especially beneficial for those who are anxious, less confident or inexperienced exercisers:

“I think getting people properly involved initially, and like [instructor 3] said before, like a month between first programme and second programme is a long time if you’re feeling a bit overwhelmed by the gym environment”. (Instructor 1)

### Skills

Participants felt that being able to perform the exercises properly was an important factor for exercise engagement. For exercises that are seen as technical, participants reported being apprehensive to engage, often because of fear of injury:

“I tried to avoid doing deadlifts in a gym because I didn’t want to injure my back”. (Participant 2)

Once the necessary skill proficiency is obtained, it is seen to facilitate a participant’s adherence to exercise without fear of injury:

“My personal trainer actually told me that I have very good control of my body so like I wasn’t really afraid of anything because I know I’ve done exercise my whole life so I wasn’t afraid of hurting myself”. (Participant 4)

Participants highlighted the important role instructors played in facilitating exercise engagement. This included that instructors were “suited to your needs” (Participant 1) and offering tailored support by “explaining things differently to different people” (Instructor 3) depending on previous knowledge and experience. This was seen to increase skill level among Healthy Minds participants:

“Having someone teach me the weights and how to use them in the correct posture was a huge thing for me because I’m a lot more confident there now”. (Participant 5)

Similar to the instructors, participants felt one instructor led session every four weeks was not frequent enough for some individuals:

The gap between it [one-to-one sessions] kind of meant that I had to self-motivate a bit and it was kind of hard to then keep it up when I only had one session. I didn’t necessarily know everything I could do and it’s good when you have more guidance. (Participant 3)

### Physical fitness and health

Participants felt that current physical fitness and health were important for continued exercise engagement. Poorer physical health and energy constraints were seen to inhibit sustained adherence to exercise:

“I had kind of physical difficulties with energy and stuff at times. Even if I wanted to [exercise], sometimes my physical health prevented that”. (Participant 1)

Recovery from exercise was also seen as a barrier for sustaining exercise:

For the next couple of days, the most I would possibly do is walking because I would be tired on that day and the next, and then wouldn’t want to do weights because I would still be aching from the session. (Participant 7)

One facilitating factor is the provision of exercises that cater for different physical abilities. For example, participants highlighted that they were more likely to engage due to the variation in exercises available. For example, utilising the fitness class offerings they gained access to as a result of being part of Health Minds:

I would have liked to have done body pump, like the weightlifting classes I used to do, but I just didn’t have the physical strength. But yoga is so gentle and it was something that I could do so I did that a lot. (Participant 1)

### Opportunity

For participants, the physical (accessibility, time) and social (social support, subjective norm) opportunities were perceived to influence exercise engagement.

#### Accessibility

Access to facilities, exercise offerings within a facility and the provision of necessary equipment were seen as important factors by participants when seeking to engage in exercise. In terms of access to facilities, external factors such as travel distance or transport and cost of gym membership were mentioned:

“My main barrier was the location of the gym and also maybe cost”. (Participant 2)

Apart from the gym, access to other offerings within a sports centre such as fitness classes were important to cater for peoples’ exercise preferences:

“It [Healthy Minds] gives them [participants] that choice of doing activities they actually want to do and enjoy”. (Instructor 1)

Therefore, participants felt that Healthy Minds increases accessibility to exercise through a cost-free 12 week membership to a range of university exercise facilities. Furthermore, participants felt that Healthy Minds allowed them to find forms of exercise that would fit their schedule and potentially lead to them sustaining this behaviour after the programme:

I could use it for free and see if it’s something that I can fit into my lifestyle so I could think about maybe financing it by myself later. So it’s quite like helpful in letting me decide if exercise is something that actually fits well into my schedule so I was really motivated because just having that access. (Participant 6)

However, when one is initially enrolled in the programme, additional guidance would ensure the full utilisation of offerings is possible:

“At the start I wasn’t quite sure what was going to happen so maybe a bit more guidance then of how to make the most of it just, maybe like a little booklet”. (Participant 5)

This point was echoed by the instructors who recommended providing additional information regarding the exercise options even beyond programme completion to facilitate sustained exercise engagement:

Like the memberships and all the options of like…I don’t know sports, what they can play or classes. I don’t know a lot of things like that. I think that would be good, like a little package. We can give our clients the leaflet. (Instructor 3)

#### Time

Time was noted as a barrier by some participants which could limit opportunities to be active:

“I think time was quite a big pressure on me and a bit of a barrier to being able to do things regularly and kind of getting in the habit of exercising really regularly”. (Participant 3)

This sample comprised of university students with academic commitments. Participants mentioned busy periods in the academic year which made exercise engagement and adherence more difficult:

“The problem was trying to carry that on [exercise] outside of the session. I mean that might have been complicated by the fact that it was when all my deadlines were due and just before exams”. (Participant 7)

Participants highlighted the benefit of being able to choose the timing of sessions and frequency of independent exercise away from the instructor. This is particularly useful for students with academic commitments:

“As a student, of course you don’t have a set timetable every week and things change and you have meetings at random times so having the ability to just chop and change and pick things is really good”. (Participant 5)

#### Social support

Participants felt one-to-one instruction helped overcome barriers by providing tailored support and answering specific exercise-related questions:

“Having the trainers kind of explain things and be there with you was really useful rather than just being plunged in on your own”. (Participant 3)

Despite having access to this support, Participant 4 mentioned that “meeting new people has always been very scary for me”, which highlights the need for instructors to be mindful to convey understanding and friendliness when working with this population:

“[The instructor] was so nice and so down to earth and so like aware of the fact that students on this programme obviously have maybe mental health issues, or you know, not as confident and things like that”. (Participant 1)

Apart from instructor support, the programme had an online forum, which has the potential to improve social opportunities, however engagement through this medium was lacking:

“It [question posted in the online forum] didn’t receive much comments or replies. It can definitely be beneficial, but I did not find it that beneficial”. (Participant 2)

Inclusion of a social aspect, allowing Healthy Minds participants to exercise together, was viewed positively by some participants. Participant 7 noted how this would give participants the chance to meet others on the programme “in the same boat” who may experience similar challenges in relation to exercise which could create a more comfortable environment. However, this option may only be suitable for those that feel confident in a group environment:

“After I know the exercises, maybe a group setting would be nicer if we have that personal trainer, like a class basically. Yeah so initially having a one-to-one would be nice so that you can make sure that you’re doing it correctly and then having a group setting would be good afterwards”. (Participant 4)

#### Subjective norm

The university’s mental health service played a prominent role by recommending exercise for wellbeing and providing participants with an opportunity to be more physically active through referral to Healthy Minds:

The counsellor sort of spoke about five ways of well-being and she was asking me which ones I felt like I was lacking which might be affecting my mental health, and one of the things she brought up was physical exercise (Participant 6).

Some participants also noted how peer influence can entice exercise-related behaviours, through observation of fellow students:

“Watching a lot of people around you take fitness seriously, working out and incorporate into their schedule makes a huge impact”. (Participant 6)

Some individuals prefer exercising with other people present. This was possible through the fitness classes at the indoor sports centre which had a positive influence on exercise engagement for some participants:

“I’m quite competitive so it would be the fact that, you know I see them [other people], and it would motivate me to do more or better”. (Participant 4)

### Motivation

Themes related to reflective (planning and routine, goal-setting) and automatic (benefits of exercising, enjoyment) motivation were seen to influence exercise engagement.

#### Planning and routine

Although it can be difficult balancing academic commitments with recreational time, participants felt that the ability to plan and prioritise exercise was important:

“I’d say because I prioritize gym, after my studies obviously and I’d like to schedule. I’d mainly go gym in the in the evenings, like maybe about 7 or 8[pm] and it was part of my routine”. (Participant 2)

Despite this, the instructors still highlighted certain challenges when planning one-to-one sessions that sometimes impact a participant’s ability to attend these sessions such as, such as travelling home, illness or holidays:

I think as well what’s tricky is when either they’re off because they’re ill, you’re off for being ill or they’re off on holiday, then you’re off on holiday, and just life happens basically. But when these things happen, it can kind of throw things out. (Instructor 2)

#### Goal-Setting

Participants mentioned that setting goals, either generally related to health or specifically related to mental health outcomes, facilitated exercise engagement:

“It’s mainly my goals and through improving my fitness and becoming healthy and stronger. That’s my main motivation and driving force for going to the gym”. (Participant 2)

“To have more calmness of mind and more confidence as well, so it was more like mental objectives rather than kind of necessarily physical”. (Participant 1)

Adapting or setting new goals were seen to facilitate sustained adherence to engaging in exercise:

“I’d also like to try and lift heavier weights, which is something I never really considered because before my priorities were to just learn how to use them. So yeah to go heavier I guess as a new goal”. (Participant 5)

As well as providing support, participants felt that the presence of an instructor provided accountability, which may serve as an enhanced motivating mechanism:

I quite liked it because there was someone else involved so that there was some kind of accountability. Because if it was just me setting the goal, then it would be too easy to kind of let it fall by the wayside. And the trainer was really friendly and really helpful so I felt kind of like I didn’t want to let them down. (Participant 7)

#### Benefits of exercising

Participants noted how the known and experienced benefits of exercise motivated them:

“Everything is connected our physical, mental, spiritual, emotional health and if we don’t exercise, then we just … all of those things are affected”. (Participant 1)

“Because I wanted results and was seeing results, I think I was more motivated to stick to the programme”. (Participant 6)

Because of this, some participants suggested the need to further promote Healthy Minds and increase awareness of how it can help students struggling with their mental health through engagement in exercise:

“I think just kind of advertising it more widely. I guess they don’t because they only have limited places. I think just making it clear to students that it is available and that is something that can support you”. (Participant 3)

However, awareness of the benefits may not always necessarily translate into exercise engagement in all cases:

“People talk about the connection between physical and mental health a lot so it’s like I knew it, but it [exercise] was hard to do”. (Participant 7)

#### Enjoyment

Participants highlighted that finding exercise they found enjoyable was important for engagement and adherence:

“I think it also depends on the type of exercise as well. Like I really enjoy spinning and I find that like not a chore at all. It’s like a really enjoyable activity”. (Participant 1)

When exercise was seen as a chore, it was perceived to impact enjoyment experienced:

“I enjoy exercise when I’m doing it but because I have this all or nothing [mindset], I turn something that should be enjoyable and should be quick and convenient into a chore when it doesn’t have to be”. (Participant 7)

## Discussion

Our research provides an understanding of the barriers and facilitators related to engagement in exercise, and specifically university-based exercise referral programmes, among students experiencing mental health difficulties. Themes aligned to the domains within the COM-B model: capability, opportunity and motivation, which helps to inform future strategies for behaviour change.

### Capability

To perform a behaviour such as exercise, an individual must have the psychological and physical capability (Michie et al., 2011). Psychological aspects found to be important included previous knowledge and experience of exercise equipment and facilities, and anxiety related to feeling judged while doing exercise. Previous research supports that a lack of knowledge and experience in such contexts and increased anxiety levels inhibit exercise engagement (Mason et al., 2019; Newsome et al., 2021; Sunesson et al., 2021). Furthermore, participants may perceive to be judged negatively when performing exercises incorrectly (Mason et al., 2019). Our research found that a university-based exercise referral scheme (ERS) alleviated such barriers through improving participants’ knowledge of exercise equipment via instruction. In addition, the face-to-face interaction and supportive environment may boost self-esteem and mental health (Katayama et al., 2020; Sunesson et al., 2021). One suggestion to further alleviate apprehension and anxiety is for increased frequency of instructor sessions during the beginning of an ERS to help overcome exercise related fears held by the participants.

Participants perceived skill level and physical health as physical factors that influence exercise engagement. Performing health-related behaviours such as exercise require sufficient skill (Newsome et al., 2021). Perceived insufficient skill can lead to fear of injury which can be a prominent concern (van Rijen & ten Hoor, 2022). In addition, physical health problems and a lack of energy inhibit exercise ability (Pelletier et al., 2017; Sunesson et al., 2021; van Rijen & ten Hoor, 2022). Strategies to alleviate such barriers include supporting skill development through structured one-to-one instruction and practice while offering a variety of exercise options to allow for different levels of physical abilities.

### Opportunity

External factors can evoke behaviour where opportunity can depend on the physical environment and/or social factors (Michie et al., 2011; Social Change UK, 2022). According to our study, time and access to facilities may influence exercise behaviour. In terms of accessibility, cost, travel and distance were specifically mentioned which aligns with previous research (Deliens et al., 2015; Griffiths et al., 2022; Sunesson et al., 2021). Time can be especially scarce for university students, especially during periods of intense academic commitments (Daskapan et al., 2006; Gómez-López et al., 2010; Griffiths et al., 2022; Silva et al., 2022). The Healthy Minds programme provided cost-free access to the university’s indoor exercise facilities which can alleviate financial barriers. However, proximity and transport can still be an issue for participants if they live far away. It is also challenging to alleviate the busy schedule faced by university students but one strategy is to offer flexibility in how participants can utilise exercise offerings to fit individual lifestyle and studies.

Our study found social support and subjective norm to be important factors for exercise engagement. Health professionals can encourage individuals to be more active (Pelletier et al., 2017). This was experienced by participants through the counselling service recommending exercise to aid mental health outcomes and facilitating exercise referral. However, peers can also influence exercise-related behaviour in the university context (Deliens et al., 2015). Findings from our study show that it can be important to offer a variety of social support, such as one-to-one sessions and access to group exercise. Both of these social aspects may help to facilitate exercise engagement and adherence in students with different levels of ability and confidence. Social support from peers has been identified as a facilitator, especially in exercise settings perceived as intimidating and can benefit mental health (Silver et al., 2019; Yorks et al., 2017). A potential suggestion for ERS in university populations is to offer opportunities to interact with others on the programme in a group setting, potentially through specific group sessions with the instructor.

### Motivation

Behaviour is driven by cognitive processes such as decision-making and emotional response which can be reflective or automatic in nature (Michie et al., 2011; Social Change UK, 2022). Reflective processes involve evaluations and plans (Michie et al., 2011). Although the long-term behaviour of participants was not examined, setting goals and scheduling exercise may help individuals maintain exercise habits beyond programme completion (Sunesson et al., 2021). In our study, goal-setting provided participants with physical and/or mental objectives to achieve which may subsequently enhance confidence related to exercise (Peddie et al., 2020). Meanwhile, being self-disciplined through incorporating exercise into one’s routine can also act as a facilitator (Deliens et al., 2015; Sims-Gould et al., 2017; van Rijen & ten Hoor, 2022). However, balancing exercise with academic commitments can still present a significant challenge for students as interrupted routines may cause increased sedentary behaviour (Newsome et al., 2021; Pellerine et al., 2022). It is important that university-based exercise schemes allow flexibility when students are planning their exercise and offer support and guidance to help generate a structure to simplify the achievement of goals (Sunesson et al., 2021).

Positive emotions towards exercise can also elicit active behaviour (Social Change UK, 2022; Willmott et al., 2021). It was seen in our study that participants engaged in exercise due to its known physical, emotional or social benefits, thereby driving autonomous motivation (Schuch & Vancampfort, 2021). However, awareness of the potential benefits of exercise may not necessarily translate into positive behavioural outcomes (Lovell et al., 2010; Newsome et al., 2021). Experiencing improvements was important for participants when exercising. However, people may leave interventions before such benefits can be obtained. Ensuring participant enjoyment when exercising, instead of viewing it as a “chore” can facilitate this engagement (Glowacki et al., 2017; van Rijen & ten Hoor, 2022). It is important for programmes to provide a variety of exercise options and allow participants autonomy when choosing an exercise they enjoy as this can encourage exercise participation (Newsome et al., 2021). A final consideration for an ERS is to provide participants with sufficient information of the programme structure and offerings at the beginning, and to provide further information of ways to stay involved in exercise after programme completion.

### Implications

According to Hettler and colleagues, a university’s function is to provide an atmosphere and environment in which students have opportunities to improve their knowledge, skills and attitudes in relation to their wellness (Hettler et al., 1980). Thus, universities can help students establish and maintain healthy lifestyle habits such as exercising that can aid their wellbeing (Hettler et al., 1980). Our research has demonstrated elements of acceptability and feasibility of a university-based exercise programme for mental health by reporting the beneficial role a university ERS can play in facilitating exercise. In addition, suggestions were identified to improve engagement and experiences in such programmes. However, it must be acknowledged that our findings are relevant to the Healthy Minds programme which brings into question the generalisability for programmes in other contexts or regions. Despite this, universities may consider implementing an ERS to support students experiencing mental health difficulties, particularly since they can assist mental health management, provide a sense of achievement, boost mood and alleviate stress (Jeftic, et al., 2023a; Lange, 2018 National Institute for Health and Care Excellence, 2022).

According to our study, the successful implementation of a mental health ERS in the university context may rely on the following components. Firstly, programmes should be flexible and individually tailored to cater for time constraints, academic commitments and physical capabilities. Secondly, access to a variety of exercise offerings will account for individual preferences and may facilitate enjoyment. Participants should be fully aware of offerings and it may be important to address financial barriers that may inhibit access to facilities. Thirdly, instructors can help individuals obtain the necessary knowledge, skills and confidence in exercise settings such as the gym. This is especially important for anxious or less experienced participants. Therefore, increasing the frequency of one-to-one instruction at the start of a programme may help participants become more comfortable in unfamiliar exercise environments. Furthermore, individuals should feel supported to attain goals throughout the entire programme. Fourthly, social opportunities could be provided where participants can interact and engage with one another. This may be particularly beneficial for those motivated to exercise with their peers. Finally, university mental health services must ensure eligible participants are aware of the existence of such programmes and facilitate referral. Considering such strategies when designing and implementing programmes to support mental health in university contexts has the potential to enhance capability, provide opportunities, and increase motivation for students to engage in exercise.

In addition to being a promising alternative treatment for mental health difficulties, promoting exercise can enhance wellbeing and thus, is a key consideration of the physical dimension of Hettler’s model (National Wellness Institute, 2023b, Lange, 2018). A state of wellness enables one to function optimally within their current environment so enabling students with mental health difficulties to engage in health-enriching behaviours such as exercise can be one tool that may help in navigating and managing life at university (National Wellness Institute, 2023a).

### Strengths and limitations

Strengths of our study include the qualitative research design which enabled rich and in-depth information to be collected from an important, at-risk population group (university students experiencing mental health difficulties), on a relatively understudied topic. Additionally, the use of triangulation, involving programme instructors helps add validity to the study findings (Cornish et al., 2014). Limitations include, that this sample was composed of participants who adhered to a university-based ERS. Therefore, research is required to gain insights from those who have dropped-out of similar ERSs, which can help identify further barriers faced by this population especially since mental ill-health may inhibit exercise participation (Marashi et al., 2021). Secondly, all participants were involved in an ERS which aimed to support students experiencing mental health difficulties within one university. Therefore, replicating similar studies in other contexts would be useful to determine the transferability of findings. Finally, we acknowledge that the sample size may be small. However, the data collected were rich and as a result of strong dialogue, provided in-depth insights relevant to the research objectives ensuring the sample held sufficient information required for the study (Malterud et al., 2016; Vasileiou et al., 2018).

## Conclusion

Our study has identified the factors influencing exercise engagement among university students with mental health difficulties participating in a university-based exercise referral scheme. To facilitate exercise engagement among students with mental health difficulties, universities may consider adopting programmes similar to Healthy Minds which promotes mental and physical health. University-based exercise programmes for mental health are likely to foster engagement and adherence if they are designed to overcome the key barriers related to exercise. This can be achieved by considering and targeting the constructs of the COM-B model (Capability, Opportunity, Motivation). The findings of our research can be used to inform future development and delivery of mental health ERS in university populations. Further research is required to enhance knowledge of this field and to ensure the successful implementation of mental health ERS in both universities and other populations.

## Supplementary Material

Supplementary File_Meaning Units.docx
