# Supplementary material for: Barriers and facilitators to engaging in a university-based exercise programme delivered to students experiencing mental health difficulties: A pilot study
Source: Cogent Ment Health. 2024 Jul 31;3(1):2380500. doi: 10.1080/28324765.2024.2380500 (PMC12443013; doi:10.1080/28324765.2024.2380500)
Supplement: Supplementary File_Meaning Units.docx [file OAMH_A_2380500_SM0944.docx]

| **COM-B construct** | **Theme identified** | **Meaning Unit** |
| --- | --- | --- |
| Capability | Experience and Knowledge | ‘You kind of feel a little bit inferior going there for the first time and using machines that you don't necessarily know what you're doing’ (Participant 1).  ‘I never really experienced any challenges because I was familiar with the gym because I’ve been going to the gym for a while. I’d say one challenge I did experience was using fitness classes because I've never really gone to fitness classes’ (Participant 2).  ‘I always did a lot of sport when I was younger so that probably helped’ (Participant 3).  ‘learning about kind of circuits were helpful because what I've been trying to do at home was mostly through guesswork’ (Participant 7).  ‘I think it is just kind of support, knowledge... you know a lot of them...Quite a lot of them have never been in the gym before. So you know it's just having that kind of base where they can kind of come to and learn I think. I think that's what quite a lot of mine have fed back to me. They've appreciated the most is that they've kind of started with no knowledge and by the time they leave the 12-week program, they actually feel like they understand what to do and why they're doing things as well in the gym’ (Instructor 1). |
|  | Anxiety | ‘I'm quite an anxious person like just socially awkward and stuff and like always meeting new people, I find that really difficult’ (Participant 1).  ‘I think I just felt a bit intimidated, a bit anxious kind of in the gym setting after not being there for quite a while’ (Participant 3).  ‘The last couple of times I went [exercising] like I still felt that anxiety about it, but I had like... over the previous time no one's batted much of an eyelid, it's not a problem. So it was getting better’ (Participant 7).  ‘I feel like people are very scared of going into the gym because they think everybody like knows everything what they're doing’ (Instructor 3).  ‘I think getting people properly involved initially, and like [instructor 3] said before, like a month between first programme and second programme is a long time if you're feeling a bit overwhelmed by the gym environment’ (Instructor 1). |
|  | Skills | ‘I tried to avoid doing deadlifts in a gym because I didn't want to injure my back’ (Participant 2).  ‘My personal trainer actually told me that I have very good control of my body so like I wasn't really afraid of anything because I know I’ve done exercise my whole life so I wasn't afraid of hurting myself’ (Participant 4).  ‘Having someone teach me the weights and how to use them in the correct posture was a huge thing for me because I’m a lot more confident there now’ (Participant 5).  ‘The gap between it [one-to-one sessions] kind of meant that I had to self-motivate a bit and it was kind of hard to then keep it up when I only had one session. I didn't necessarily know everything I could do and it's good when you have more guidance’ (Participant 3).  ‘because we know what we're doing as well it is probably giving them a little bit more support just because of the knowledge and if they do something wrong we can correct their technique’ (Instructor 3). |
|  | Physical Fitness and Health | ‘I had kind of physical difficulties with energy and stuff at times. Even if I wanted to [exercise], sometimes my physical health prevented that’ (Participant 1).  ‘For the next couple of days, the most I would possibly do is walking because I would be tired on that day and the next, and then wouldn't want to do weights because I would still be aching from the session’ (Participant 7).  ‘I would have liked to have done body pump, like the weightlifting classes I used to do, but I just didn't have the physical strength. But yoga is so gentle and it was something that I could do so I did that a lot’ (Participant 1).  ‘But for the next couple of days, the most I would possibly do is walking because I would be tired on that day and the next and then wouldn't want to do weights because I would still be aching from the session’ (Participant 7).  ‘I ask them... this is for my benefit... I ask them for what they're kind of exercise or sports kind of history is just that I know where to base things at the start’ (Instructor 1). |
| Opportunity | Accessibility | ‘My main barrier was the location of the gym and also maybe cost’ (Participant 2).  ‘I could use it for free and see if it's something that I can fit into my lifestyle so I could think about maybe financing it by myself later. So it's quite like helpful in letting me decide if exercise is something that actually fits well into my schedule so I was really motivated because just having that access’ (Participant 6).  ‘The access to the facilities for me was huge because I didn't know what I wanted to do and also how to afford it’ (Participant 5).  ‘I like that I got access to the gym and the pool and everything. So I wouldn't have been able to afford that if I didn't do the Healthy Minds’ (Participant 3).  ‘It [Healthy Minds] gives them [participants] that choice of doing activities they actually want to do and enjoy’ (Instructor 1). |
|  | Time | ‘I think time was quite a big pressure on me and a bit of a barrier to being able to do things regularly and kind of getting in the habit of exercising really regularly’ (Participant 3).  ‘The problem was trying to carry that on [exercise] outside of the session. I mean that might have been complicated by the fact that it was when all my deadlines were due and just before exams’ (Participant 7).  ‘As a student, of course you don't have a set timetable every week and things change and you have meetings at random times so having the ability to just chop and change and pick things is really good’ (Participant 5).  ‘I had my moments of being quite lazy I think the first year of my masters and the course was just so intense and I had a part time job at the time, so I didn't do any exercise as I just didn't have the time really, but when I have more time on my hands I did’ (Participant 1).  ‘Lack of knowledge, time, stress, anxiety. Yeah basically those are probably the top things [barriers]’ (Instructor 2). |
|  | Social Support | ‘Having the trainers kind of explain things and be there with you was really useful rather than just being plunged in on your own’ (Participant 3).  ‘[The instructor] was so nice and so down to earth and so like aware of the fact that students on this programme obviously have maybe mental health issues, or you know, not as confident and things like that’ (Participant 1).  ‘It [question posted in the online forum] didn't receive much comments or replies. It can definitely be beneficial, but I did not find it that beneficial’ (Participant 2).  ‘I think she [the instructor] just listened to everything that I had to say. She talked me through all of the machines really slowly and gave me like a really set plan for every session which I really appreciated because that was my struggle, was the plan and she actually took the time to work through any like posture problems that I had. And when I was doing it right, she was like really reassuring because actually because I didn't think I was doing it right at all but turns out, you know, it was fine and yeah she was just very friendly’ (Participant 5).  ‘I always try to compliment that they're doing things right. I'll really try and big it up because then I think that will then help with them believing in themselves and know that they actually can do something’ (Instructor 1). |
|  | Subjective Norm | ‘The counsellor sort of spoke about five ways of well-being and she was asking me which ones I felt like I was lacking which might be affecting my mental health, and one of the things she brought up was physical exercise’ (Participant 6).  ‘Watching a lot of people around you take fitness seriously, working out and incorporate into their schedule makes a huge impact’ (Participant 6).  ‘I’m quite competitive so it would be the fact that, you know I see them [other people], and it would motivate me to do more or better’ (Participant 4).  ‘I hadn't heard about [Healthy Minds] until my final year and I had a couple of like counselling sessions with student health. And then it was mentioned to me in one of those’ (Participant 3).  ‘I'd say I was familiar with the benefits of physical activity, such as the social aspects, obviously from playing football, going to gym with my friends’ (Participant 2). |
| Motivation | Planning and Routine | ‘I'd say because I prioritize gym, after my studies obviously and I’d like to schedule. I’d mainly go gym in the in the evenings, like maybe about 7 or 8[pm] and it was part of my routine’ (Participant 2).  ‘Just kind of getting a routine of going again and it just made me like think of it a bit more and be like Oh, I could just go for a swim today or whatever yeah definitely. Became a bit more of a habit’ (Participant 3).  ‘I find I’ve scheduled in my gym days in my schedule’ (Participant 4).  ‘I think the main thing is helping with the organizational side. I think for anyone who's reaching out to this is because they're a bit muddled, don't really know what to do and find it hard to, I guess… this is just my experience but find it difficult to go and do it themselves. So I think any extra guidance that it can provide is really beneficial and that structure was a huge thing for me’ (Participant 5).  ‘I think as well what's tricky is when either they’re off because they're ill, you're off for being ill or they're off on holiday, then you're off on holiday, and just life happens basically. But when these things happen, it can kind of throw things out’ (Instructor 2). |
|  | Goal-Setting | ‘It’s mainly my goals and through improving my fitness and becoming healthy and stronger. That's my main motivation and driving force for going to the gym’ (Participant 2).  ‘To have more calmness of mind and more confidence as well, so it was more like mental objectives rather than kind of necessarily physical’ (Participant 1).  ‘I’d also like to try and lift heavier weights, which is something I never really considered because before my priorities were to just learn how to use them. So yeah to go heavier I guess as a new goal’ (Participant 5).  ‘I quite liked it because there was someone else involved so that there was some kind of accountability. Because if it was just me setting the goal, then it would be too easy to kind of let it fall by the wayside. And the trainer was really friendly and really helpful so I felt kind of like I didn't want to let them down’ (Participant 7).  ‘I ask them...Oh kind of if they've got any goals and I don't mean specific like body goals or anything like that... just generally you know they want to get fitter because they've got this or they just want to be able to play five a side football. You know anything really health related, if there's anything they really want to achieve’ (Instructor 1). |
|  | Benefits of Exercising | ‘Everything is connected our physical, mental, spiritual, emotional health and if we don't exercise, then we just…all of those things are affected’ (Participant 1).  ‘Because I wanted results and was seeing results, I think I was more motivated to stick to the programme’ (Participant 6).  ‘People talk about the connection between physical and mental health a lot so it's like I knew it, but it [exercise] was hard to do’ (Participant 7).  ‘Yeah I think once I see kind of the benefits of it, then it feels more enjoyable and like once you kind of get the progression that's very motivating’ (Participant 3).  ‘Yeah I think so, and then some people think it's really sort of helped them when they really...You know I can think of other examples of people when they've been really struggling. It's been like a sort of like a lifeline for them to come out of the where they're living and just have somewhere else to go to, where they can kind of...They feel better after they've trained so yeah’ (Instructor 2). |
|  | Enjoyment | ‘I think it also depends on the type of exercise as well. Like I really enjoy spinning and I find that like not a chore at all. It's like a really enjoyable activity’ (Participant 1).  ‘I enjoy exercise when I’m doing it but because I have this all or nothing [mindset], I turn something that should be enjoyable and should be quick and convenient into a chore when it doesn't have to be’ (Participant 7).  ‘So I do enjoy exercising and I enjoy sport and being active and so I can separate that from the gym so I do enjoy exercise and sport’ (Participant 2).  ‘I enjoy these weight classes because they kind of hit every aspect and the short amount of time. So probably like HIIT and weights’ (Participant 5).  ‘And I can put it off like easily but once I'm there, I really enjoy it and like after I’m done working out, I feel really good about it. So if I have had a good workout session I’m motivated to go again and again and again’ (Participant 6). |
